# Successfully Deflating a Stubborn Foley Catheter Balloon With Mineral Oil

**DOI:** 10.7759/cureus.107794

**Published:** 2026-04-27

**Authors:** Vijay Reddy, Abigail Thompson

**Affiliations:** 1 Emergency Medicine, Augusta University Medical College of Georgia, Augusta, USA

**Keywords:** benign prostatic hyperplasia, foley catheter, mineral oil, silicone balloon, urinary retention

## Abstract

Indwelling Foley catheters are commonly used in the healthcare setting for urinary tract management both acutely and chronically, but are associated with multiple complications that require emergency department intervention. We report an 81-year-old male patient with a history of benign prostatic hyperplasia who presented to the emergency department with a nondeflating catheter balloon and the successful removal of the catheter after use of mineral oil to deflate the balloon after failure of other conservative measures that are considered first-line in the medical literature. The patient was subsequently able to pass a spontaneous voiding trial and was discharged from the emergency department in stable condition. This case highlights a practical approach to consider in the case of nondeflating Foley catheter balloons that can be used in the home health, ambulatory care, and emergency department settings.

## Introduction

The use of balloon-inflated urinary catheters in healthcare started with the invention of the Foley catheter by Dr. Frederic Foley, who first described such a catheter in 1929 [1]. These catheters were initially used for hemostasis after prostatectomy, but are now clinically indicated for many different pathologies, both in the acute care setting as well as for chronic management of conditions such as neurogenic bladder, bladder outlet obstruction, and urinary incontinence. It is estimated that 100 million urinary catheters are used yearly [2]. A systematic review of nursing home patients showed that as many as 9.3% of nursing home residents in the United States have chronic indwelling catheters, with prevalence increased in male patients and seeming to increase with increasing age [3]. This, of course, does not include the number of patients who have chronic indwelling catheters but are managed at home or in other settings.

The use of chronic Foley catheters leads to emergency department visits related to multiple complaints. There are many known complications of chronic Foley catheter usage, the most concerning and studied of which are catheter-associated urinary tract infections (CAUTIs). Patients also at times will present to the ED due to bladder spasms, leakage, blockage, trauma, bladder stones, difficulty with Foley replacement, need for irrigation, and sometimes even the descriptively named "purple urine bag syndrome" [2]. These lead to increased emergency department utilization, urologic consultations, and sometimes hospital admissions. Therefore, the management of Foley catheters in the emergency department is a necessary skill for all emergency physicians to learn.

In this article, we present another potential complication of a Foley catheter requiring emergency department management, the nondeflating Foley balloon.

## Case presentation

An 81-year-old African-American male, currently on hospice, with a history of gastrointestinal stromal tumor, heart failure with preserved ejection fraction, benign prostatic hyperplasia, diabetes mellitus type II, and severe obesity, presented to the ED with a complaint of discomfort related to the presence of a Foley catheter. His home hospice nurse attempted to deflate the balloon to remove the catheter and was unable to. An attempt was also made to cut the catheter to attempt to deflate the balloon, and this was also unsuccessful. The patient was then sent to the emergency department for management.

**Figure 1:**
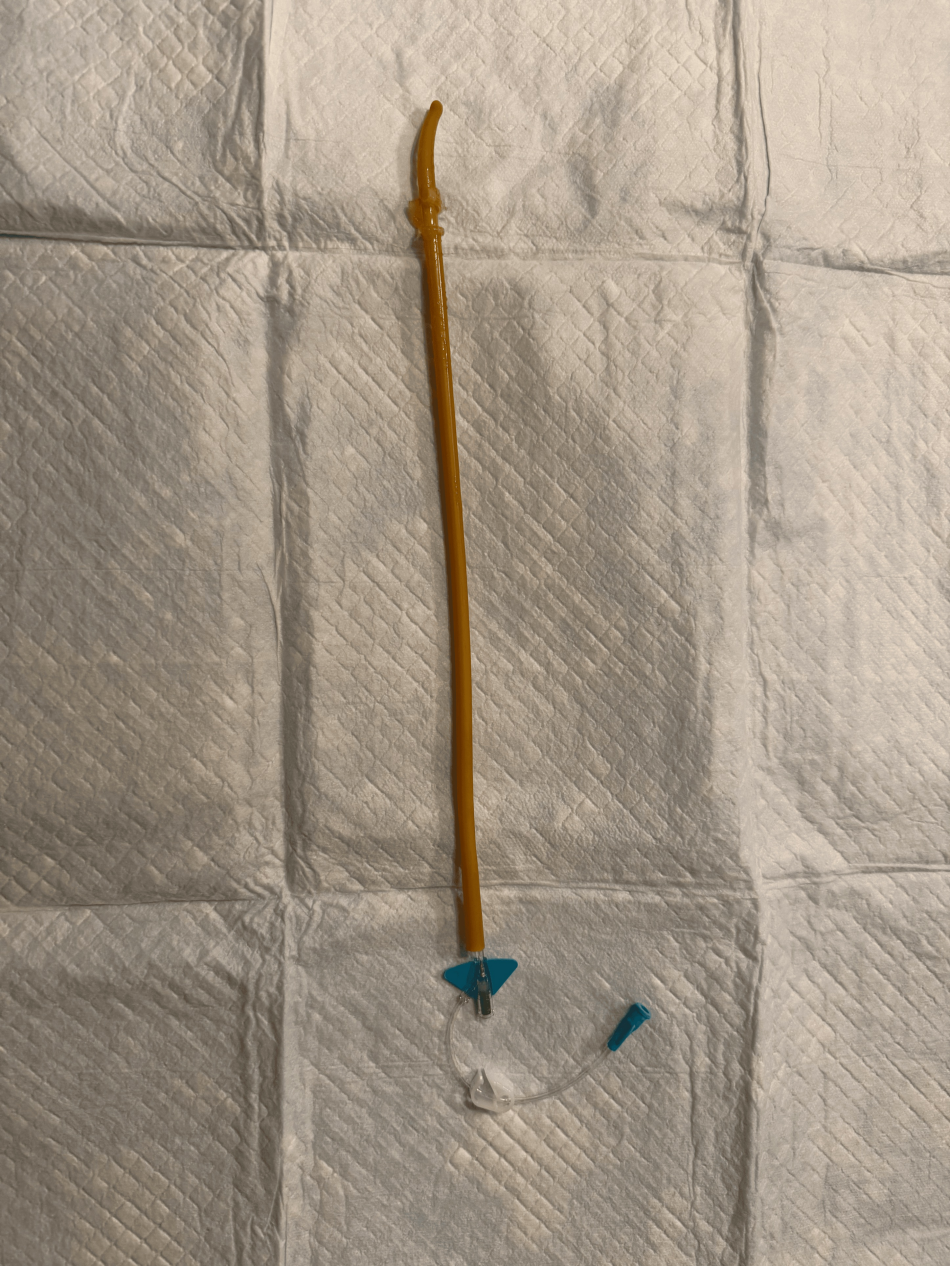
Foley catheter after balloon rupture, with 22-gauge angiocatheter in balloon inflation channel

The patient presented to the emergency department mildly hypertensive, but otherwise had no vital sign abnormalities. He otherwise had no complaints other than discomfort related to his urinary retention. Attempts to aspirate the balloon inflation lumen using an angiocatheter in the lumen to relieve the obstruction were unsuccessful. Numerous attempts were made to use a guidewire to attempt to puncture the balloon, but there was no guidewire available to us that would be both long enough and thin enough to pass through the balloon port on the Foley catheter. A 22-gauge angiocatheter and a syringe were used to instill 10 mL of mineral oil into the balloon port, with some pressure needed to instill the mineral oil due to light resistance. There was no immediate change, but after about 10 minutes, a popping sound was heard, and the Foley catheter was easily able to be removed (Figure 1). The patient reported resolution of his discomfort, was able to spontaneously void, received 1 gram of intramuscular ceftriaxone for prophylaxis, declined Foley reinsertion, and was subsequently discharged from the emergency department.

## Discussion

Mineral oil has several clinical indications, being most commonly used for the treatment of constipation, either in an oral formulation as a laxative or for use as a rectal enema. Due to this, it is readily available in most healthcare settings and available over the counter for purchase. It also has nonclinical utility as a lubricating fluid. Notably, the use of mineral oil for the lubrication of gastrostomy balloons on insertion is contraindicated, as this would lead to loss of integrity of the balloon. In much the same way that mineral oil was useful in this case, this method may also be useful for other medical devices that rely on inflated balloons, such as those in a gastrostomy. 

There are many reasons to remove a Foley catheter, such as for routine exchange, lack of drainage, or patient preference. Nondeflating Foley catheter balloon is a known complication, caused by such things as debris clogging the balloon inflation channel, the fluid in the balloon having crystallized (usually because a fluid other than water was used), or a defect in the valve mechanism [4]. Methods to resolve a nondeflating Foley catheter balloon have been discussed in the literature, with Shapiro et al. suggesting an algorithm favoring use of a guidewire prior to suggesting many other potential methods, such as cutting the balloon port and use of a central venous catheter, before consideration of chemical dissolution or mechanical puncture (be it transurethral, percutaneous, or endoscopic) [5]. Suprapubic puncture of the balloon has been suggested in the literature [6]. A case report described successfully using a 20-gauge needle in the balloon inflation channel to pop the balloon [7]. Another case report suggested the use of a trochar needle for deflation of a balloon in a nephrostomy tube, though there was some external damage to the renal pelvis in this case [8]. Further methods of puncture or aspiration of the balloon described in the literature include transvaginal or intraurethral puncture in females [9-10], a transrectal approach noted in a case report in dogs [11], and approaches involving needle guidance by ultrasound, or use of cystogram [12]. Other dissolving agents, such as chloroform, have also been described [13], though this would be a less readily available substance. The use of the mineral oil method described in this case report allows this procedure to be performed with less risk of complications related to local trauma.

When considering dissolution or puncture of a Foley catheter balloon, some consideration can be given to the material the balloon is composed of. Both latex and silicone balloons are susceptible to dissolution in mineral oil. While there has not been a direct comparison of the use of mineral oil in these two types of indwelling catheter balloons, it has been previously documented in the literature that the overinflation of latex balloons can cause residual fragments in the bladder, while silicone balloons were not prone to this [14]. This study did not investigate the effects of balloon dissolution, but did note that if a balloon was punctured without being overinflated, neither latex nor silicone balloons left any fragments. Attention should be given to follow-up if it is suspected that a significant fragment was left behind in the bladder after a dissolution attempt using this method, though it is worth noting that this would also be true for most other methods described in the literature for a nondeflating Foley balloon (such as dissolution, puncture, or overinflation). Further research into the risk of balloon fragments or other local complications after mineral oil use should be considered.

**Figure 2:**
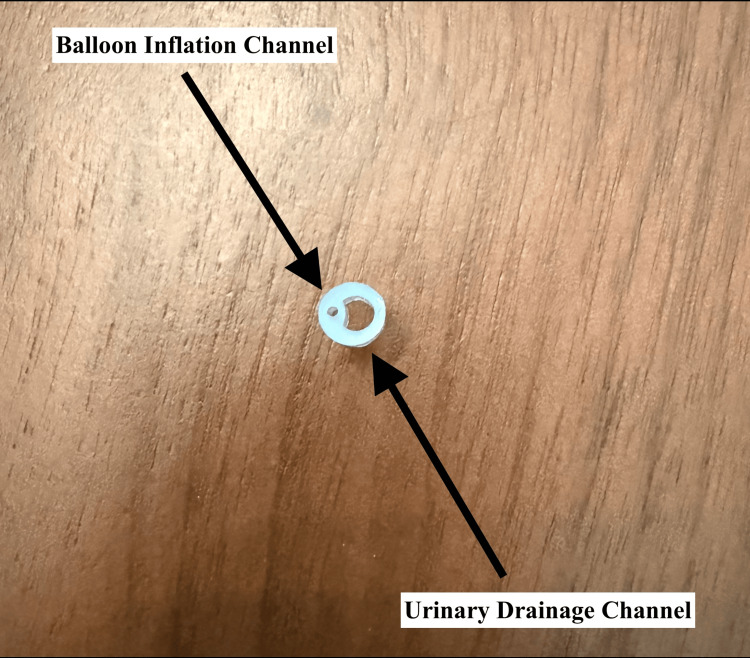
Foley catheter in cross section

In the situation that the Foley catheter is cut or fractured distal to the ports, using a small-bore IV catheter, as seen in Figure 1, inserted into the balloon inflation channel (situated next to the urinary drainage channel, as seen in Figure 2) allows access to the balloon to attempt to withdraw the balloon’s contents or to instill mineral oil into the balloon using a syringe. Often, an early management step for a nondeflating balloon is to cut the valve stem of the balloon inflation channel [4], and so the use of an IV catheter in the balloon inflation channel would be a requirement once the valve has been cut. Notably, there is a fair amount of pressure required to instill the mineral oil due to its viscosity, and this should be expected when the procedure is performed.

There is no clear evidence in the literature supporting the use of antibiotics after the successful dissolution of a Foley balloon, but consideration was given to the potential for the introduction of microorganisms after the Foley balloon's contents were disseminated into the bladder. A systematic review did show a reduction in post-catheterization UTIs in patients who received short-term indwelling catheters, but suggested that patient selection may be important to mitigate complications and promote antibiotic stewardship [15]. Antibiotic prophylaxis has been used after a Foley balloon rupture previously [16], though there is no clear evidence, and it seems to be provider-dependent. The need for postprocedural antibiotics after the rupture of a Foley catheter balloon, especially in a chronic indwelling catheter, would be an interesting future topic for study.

## Conclusions

The nondeflating Foley catheter balloon is a well-established complication that can be managed in the emergency department, ambulatory care, or home setting without the need for instrumentation or urology consultation using mineral oil. Mineral oil is safe, well-tolerated, and readily available. This method is a useful tool in the management of nondeflating Foley catheter balloons and may help to prevent unnecessary patient transfers, consultations, hospitalizations, and invasive procedures in some cases, and may also be useful for other medical devices that use silicone or latex balloons.
